# Allogeneic hematopoietic stem cell transplantation is associated with improved survival compared with chemotherapy in patients with acute myeloid leukemia harboring favorable-risk genetic features at initial diagnosis: a single-center retrospective study

**DOI:** 10.3389/fimmu.2026.1825310

**Published:** 2026-09-15

**Authors:** Rujiao Dong, Lihua Wu, Chunling Zhao, Xianling Chen, Xiaohong Yuan, Zhijuan Zhu, Xiaofan Li, Nainong Li

**Affiliations:** 1 Fujian Institute of Hematology, Fujian Provincial Key Laboratory on Hematology, Fujian Medical University Union Hospital, Fuzhou, China; 2 Translational Medicine Center on Hematology, Fujian Medical University, Fuzhou, China

**Keywords:** acute myeloid leukemia, allogeneic hematopoietic stem cell transplantation, chemotherapy, favorable-risk genetic features at initial diagnosis, measurable residual disease, relapsed/refractory leukemia

## Abstract

**Background:**

Acute myeloid leukemia (AML) harboring favorable-risk genetic features at initial diagnosis (FR-AML) commonly achieves remission after chemotherapy, but the optimal post-remission strategy remains controversial. This study compared allogeneic hematopoietic stem cell transplantation (allo-HSCT) with chemotherapy and explored prognostic factors.

**Methods:**

We retrospectively analyzed 190 patients with FR-AML treated at Fujian Medical University Union Hospital between 2012 and 2022. Patients were stratified into the chemotherapy (n = 118) and allo-HSCT (n = 72) groups according to their post-induction treatment. Survival outcomes, including overall survival (OS), leukemia-free survival (LFS), cumulative incidence of relapse (CIR), and non-relapse mortality (NRM), were assessed using Kaplan–Meier and competing-risk analyses. Multivariate Cox regression identified independent prognostic factors.

**Results:**

At 3 years, the allo-HSCT group had higher OS (86.0% vs. 61.3%, *P* < 0.0001) and LFS (59.2% vs. 49.3%, *P* = 0.039) than the chemotherapy group, with lower CIR (3.8% vs. 33.6%, *P* < 0.0001). Stratified analyses showed that allo-HSCT was associated with improved 3-year OS compared with chemotherapy alone in CR1-AML and R/R AML patients. In CR1-AML, the 3-year OS was 92.8% versus 73.6% (*P* = 0.0099), whereas in R/R AML, it was 73.5% versus 26.6% (*P* = 0.00015). By age, outcomes were comparable among patients aged <20 years, whereas allo-HSCT was associated with better survival in those aged 20–60 years. Among 66 transplanted patients with measurable residual disease (MRD) data, 3-year OS did not differ significantly between MRD-positive and MRD-negative groups (86.0% vs. 85.6%, *P* = 0.35).

**Conclusions:**

Allo-HSCT was associated with improved survival and reduced relapse in FR-AML. This benefit extended beyond patients transplanted in CR1 to those who later develop R/R disease, in whom salvage allo-HSCT confers a marked survival advantage over chemotherapy. Deferred transplantation may remain feasible for selected patients when a suitable donor is available. Prospective studies are warranted to validate these findings and optimize post-remission treatment strategies in this population.

## Introduction

Acute myeloid leukemia (AML) is a heterogeneous clonal malignancy that originates from hematopoietic stem cells (1, 2) and is the most common type of acute leukemia in adults, accounting for approximately 80% of cases in this population (3). Despite advances in understanding the molecular heterogeneity and pathogenesis of AML (4), its prognosis remains poor, with an estimated survival rate below 30% for adult patients (5). Traditionally, cytogenetic profiles have guided the prognostic stratification before treatment (6). More recently, specific genetic mutations have been identified as additional prognostic markers (7). According to the 2022 European LeukemiaNet (ELN) risk stratification system (8), AML patients are classified into adverse-, intermediate-, and favorable-risk groups, which has important significant implications for prognosis and treatment selection (9, 10).

The “7 + 3” induction chemotherapy regimen, which combines cytarabine with anthracyclines, achieves hematologic remission in most newly diagnosed AML patients (11–13). However, relapse risk remains considerable and is influenced by age, cytogenetic and molecular characteristics, measurable residual disease (MRD) status after induction, and post-remission therapy (14, 15). Therefore, consolidation chemotherapy and hematopoietic stem cell transplantation (HSCT) remain critical strategies for extending survival in patients with AML (16, 17).

Over the past two decades, allogeneic HSCT (allo-HSCT) has become an important component of AML treatment. The decision to proceed to transplantation is generally based on a careful risk-benefit analysis (18) For patients with AML harboring favorable-risk genetic features at initial diagnosis, current guidelines recommend consolidation chemotherapy as the preferred post-induction treatment (19). Nonetheless, emerging studies indicate that autologous or allogeneic HSCT may offer survival advantages for this subgroup (20, 21), maintaining the debate over the optimal post-induction therapy. For instance, one study reported that in favorable-risk AML with double-mutant CEBPA, both autologous and allogeneic HSCT provided better survival outcomes than chemotherapy (22), whereas another study found similar survival rates between chemotherapy and allo-HSCT for the same subtype (23).

In this study, we retrospectively analyzed patients with AML harboring favorable-risk genetic features at initial diagnosis (FR-AML) treated at our institution. We compared the efficacy of allo-HSCT with chemotherapy and to explore the optimal timing for transplantation in this patient population.

## Methods

### Patients

Between 2012 and 2022, we screened 210 consecutive patients with AML harboring favorable-risk genetic features at initial diagnosis at Fujian Medical University Union Hospital, ultimately including 190 patients in the analysis. Patients were assigned to the chemotherapy (CMT) group and the allo-SCT group according to their final treatment modality. The inclusion criteria were as follows: (1) age ≤ 60 years, (2) available cytogenetic and mutation-related data, and (3) favorable-risk classification at initial diagnosis according to 2022 ELN criteria. The exclusion criteria were as follows: (1) acute promyelocytic leukemia, (2) lost to follow-up prior to receiving at least two cycles of chemotherapy and (3) autologous stem cell transplantation. The treatment flowchart is shown in **Figure 1**.

**Figure 1:**
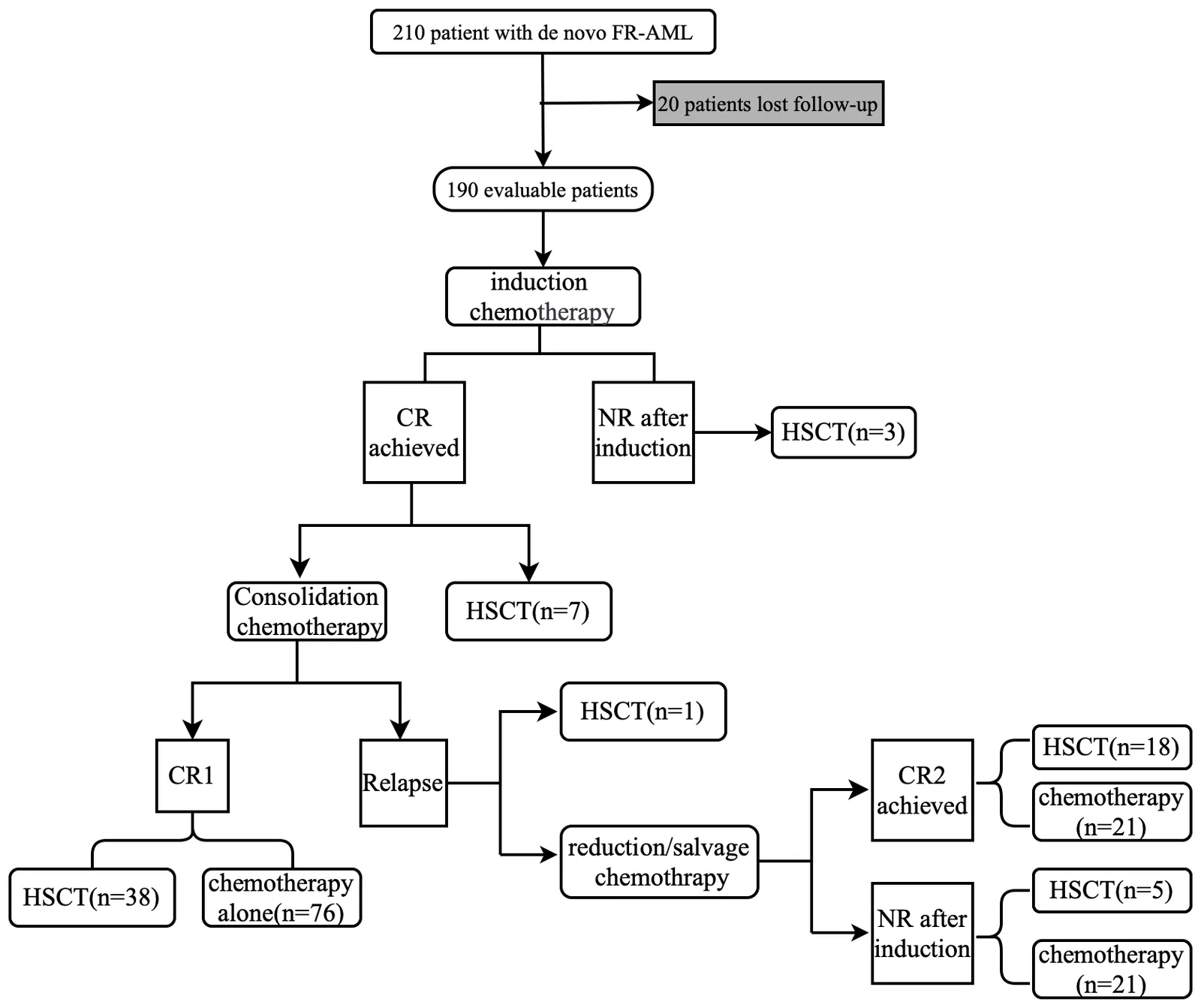
The treatment flowchart.

### Treatment

Post-induction chemotherapy was administered according to institutional practice and individualized clinical decision-making. Most patients received high-dose cytarabine-based regimens, with or without anthracyclines. Other regimens included intermediate-dose cytarabine and HAG therapy (homoharringtonine plus low-dose cytarabine).

In our cohort, allo-HSCT was considered in selected patients based on dynamic treatment response and individualized clinical assessment. Although most patients with favorable-risk AML who achieved CR1 were managed with chemotherapy-based post-remission therapy, allo-HSCT was also considered in selected CR1 patients with a suboptimal molecular response, defined as a <3-log reduction in the level of a leukemia-associated molecular marker after three cycles of chemotherapy. In addition, when a suitable donor was available, a strong and clearly expressed patient preference for transplantation was also incorporated into the decision-making process. Among patients who underwent allo-HSCT in CR1, 23 had no identified molecular-response or MRD-related risk factors and proceeded to transplantation primarily on the basis of patient preference. In general, referral for allo-HSCT was made for patients who failed to achieve CR1, experienced relapse, achieved a second or subsequent remission after relapse, had inadequate or persistent MRD after treatment. The final decision regarding transplantation also took into account donor availability, patient preference, physician judgment, and the patient’s overall clinical condition.

Maintenance therapy was not routinely administered after chemotherapy alone, although 4 of 118 (3.39%) patients received venetoclax as maintenance therapy. In the allo-HSCT group, post-transplant maintenance therapy was routinely recommended in our center according to institutional practice and clinical judgment, with interferon(0.3 MIU twice weekly for 3–6 months)generally used as the standard approach and hypomethylating agents considered for patients who were unable to tolerate interferon. 46 of 72 (63.89%) patients had clearly documented post-transplant maintenance therapy in the available records.

### Measurable residual disease assessment

MRD was assessed using multiparameter flow cytometry (MFC) and/or molecular assays according to the disease-specific abnormality identified at diagnosis. MFC-based MRD positivity was defined as residual leukemic cells at a frequency>10^-4^, based on the detection of a leukemia-associated immunophenotype. RUNX1::RUNX1T1 and CBFB::MYH11 fusion transcripts were quantified by real-time quantitative PCR, with MRD positivity defined as a fusion transcript-to-reference gene ratio ≥0.01%. NPM1 and CEBPA mutations were monitored by digital PCR, with MRD positivity defined as a mutant allele fraction ≥0.01%. Detailed MRD detection methods and cutoff values are summarized in **Supplementary Table 1**.

### Endpoints and definitions

The primary endpoint was the 3-year overall survival (OS). Secondary endpoints included leukemia-free survival (LFS), cumulative incidence of relapse (CIR), and non-relapse mortality (NRM). Complete remission (CR) was defined as <5% blasts in bone marrow, absence of leukemic blasts in peripheral blood or extramedullary sites, and recovery of blood counts. Relapse was defined by morphologic evidence of leukemia in bone marrow, peripheral blood, or extramedullary sites. OS was calculated from diagnosis to death from any cause or was censored at the last follow-up; in the R/R AML cohort, OS was calculated from the date of relapse to death from any cause or was censored at the last follow-up. LFS was defined as the interval from diagnosis to relapse or death. NRM was defined as death without relapse.

### Statistical analysis

Normality was assessed with the Shapiro-Wilk test. Non-normally distributed continuous variables were presented as medians (interquartile ranges) and compared using the Mann-Whitney U test. Categorical variables were compared with the Chi-square test. OS and LFS were estimated using the Kaplan-Meier method and compared with the Log-rank test. Cumulative incidence curves were generated in a competing risk framework, with relapse treated as a competing event for NRM death without relapse treated as a competing event for CIR.

To further reduce measured confounding arising from baseline imbalance between the allo-HSCT and chemotherapy groups, a propensity score–based inverse probability of treatment weighting (IPTW) analysis was performed. Propensity scores for receiving allo-HSCT versus chemotherapy were estimated using a multivariable logistic regression model incorporating the following baseline covariates: age, white blood cell count at diagnosis, number of cycles required to achieve complete remission (CR), number of chemotherapy cycles, relapsed/refractory status, and molecular abnormalities. Stabilized weights were calculated and truncated at the 1st and 99th percentiles to reduce the influence of extreme weights. Covariate balance before and after weighting was assessed using standardized mean differences (SMDs), with an SMD < 0.1 considered indicative of adequate balance. Weighted Cox proportional hazards models were then used to evaluate the association between treatment group and survival outcomes.

To minimize immortal time bias, a landmark analysis was conducted using 3 months after relapse diagnosis as the landmark time point. Patients who were alive at 3 months were eligible for this analysis. The transplantation group included patients who underwent transplantation within 3 months after relapse diagnosis, as well as those with a documented intent to undergo transplantation during this period, regardless of whether transplantation was subsequently performed. Patients who did not meet these criteria were classified into the chemotherapy group. Survival was measured from the landmark time point onward. For multivariable Cox regression analysis, variables were selected based on both clinical relevance and univariate analysis results. And the following variables were included in the multivariable analysis: treatment modality, age, white blood cell (WBC) count, cycles to CR, number of chemotherapy cycles, and disease status. Cox proportional hazards models, and hazard ratios (HRs) with 95% confidence intervals (CIs) were reported.

All statistical analyses were two-sided, with significance set at p = 0.05. Analyses were conducted using R software (version 4.2.2).

## Results

### Patient demographics and treatment characteristics

Among the 190 patients included in this study, 72 underwent allo-HSCT and 118 received chemotherapy alone. The median age in the allo-HSCT group was significantly lower than that in the chemotherapy group [31.0 years (range, 2-60) vs. 41.0 years (range, 2-60), respectively; *P* = 0.012]. Except for age, cytogenetic findings, and the total number of chemotherapy cycle, baseline characteristics were balanced between the groups. These characteristics included sex distribution, white blood cell count, hemoglobin level, platelet count, peripheral blood blast percentage, lactate dehydrogenase level, induction chemotherapy regimen, the proportion of patients with relapsed/refractory leukemia, and the proportion of patients who required two cycles to achieve CR (**Table 1**).

**Table 1:** The demographics and treatment characteristics of patients.

Characteristics	Allo-SCT(n=72)	CMT(n=118)	P value
Age, median [range], years	31.0 [2-60]	41.0 [2-60]	0.012
Male, n (%)	34 (47.2%)	48 (40.7%)	0.464
R/R AML, n (%)	27 (37.5%)	42 (35.6%)	0.913
Molecular abnormalities, n (%):			0.002
RUNX1::RUNX1T1	30 (41.7%)	45 (38.1%)	
CBFβ::MYH11	11 (15.3%)	30 (25.4%)	
NPM1 mutation	7 (9.72%)	27 (22.9%)	
CEBPA bZip in-frame mutation	24 (33.3%)	16 (13.6%)	
WBC, median [range], 10^9^/L	22.8 [2.69-512]	16.7 [0.57-268.3]	0.141
Neutrophils, median [range], %	55.0 [2.0-97.0]]	49.0 [5.0-99.0]	0.142
Hemoglobin, median [range], g/L	76.5 [34.0-126.0]	73.0 [32.0-171.0]	0.312
Platelet, median [range], 10^9^/L	27.5 [5.0-205.0]	35.0 [3.0-638.0]	0.162
LDH, median [range], mmol/L	473 [105-1957]	473 [119-6951]	0.671
First induction,n (%)			0.217
IA	57 (79.2%)	88 (74.6%)	
DA	6 (8.33%)	5 (4.24%)	
FLAG-IDA	6 (8.33%)	21 (17.8%)	
Others	3 (4.17%)	4 (3.39%)	
Cycles to CR, n (%)			0.125
≤2	59 (81.9%)	107 (90.7%)	
>2	13 (18.1%)	11 (9.32%)	
Cycles of chemotherapy, median [range]	4 [1-23]	7[2-36]	<0.001
ECOG:			0.054
<2	55 (76.4%)	104 (88.1%)	
≥2	17 (23.6%)	14 (11.9%)	
Comorbidity:			1.000
No	59 (81.9%)	97 (82.2%)	
Yes	13 (18.1%)	21 (17.8%)	

R/R, relapsed/refractory; WBC, White Blood Cell; IA, Idarubicin + Cytarabine; DA, Daunorubicin + Cytarabine; FLAG-IDA, Fludarabine + Cytarabine + G-CSF + Idarubicin; CR, complete remission.

### Outcomes

At 3 years, OS was significantly higher in the allo-SCT group than in the chemotherapy group (86.0% vs. 61.3%; P < 0.0001, **Figure 2A**). Similarly, 3-year LFS was 59.2% in the allo-SCT group and 49.3% in the chemotherapy group (P = 0.039; **Figure 2B**). For NRM, 35 patients in the chemotherapy group died without relapse, corresponding to a 3-year rate of 26.9%, whereas 11 patients in the allo-SCT group died without relapse, corresponding to a 3-year NRM of 12.8% (P = 0.012; **Figure 2C**). 3-year CIR in the chemotherapy group is 33.6%, compared with a significantly lower CIR of 3.8% in the allo-SCT group (P < 0.0001, **Figure 2D**).

**Figure 2:**
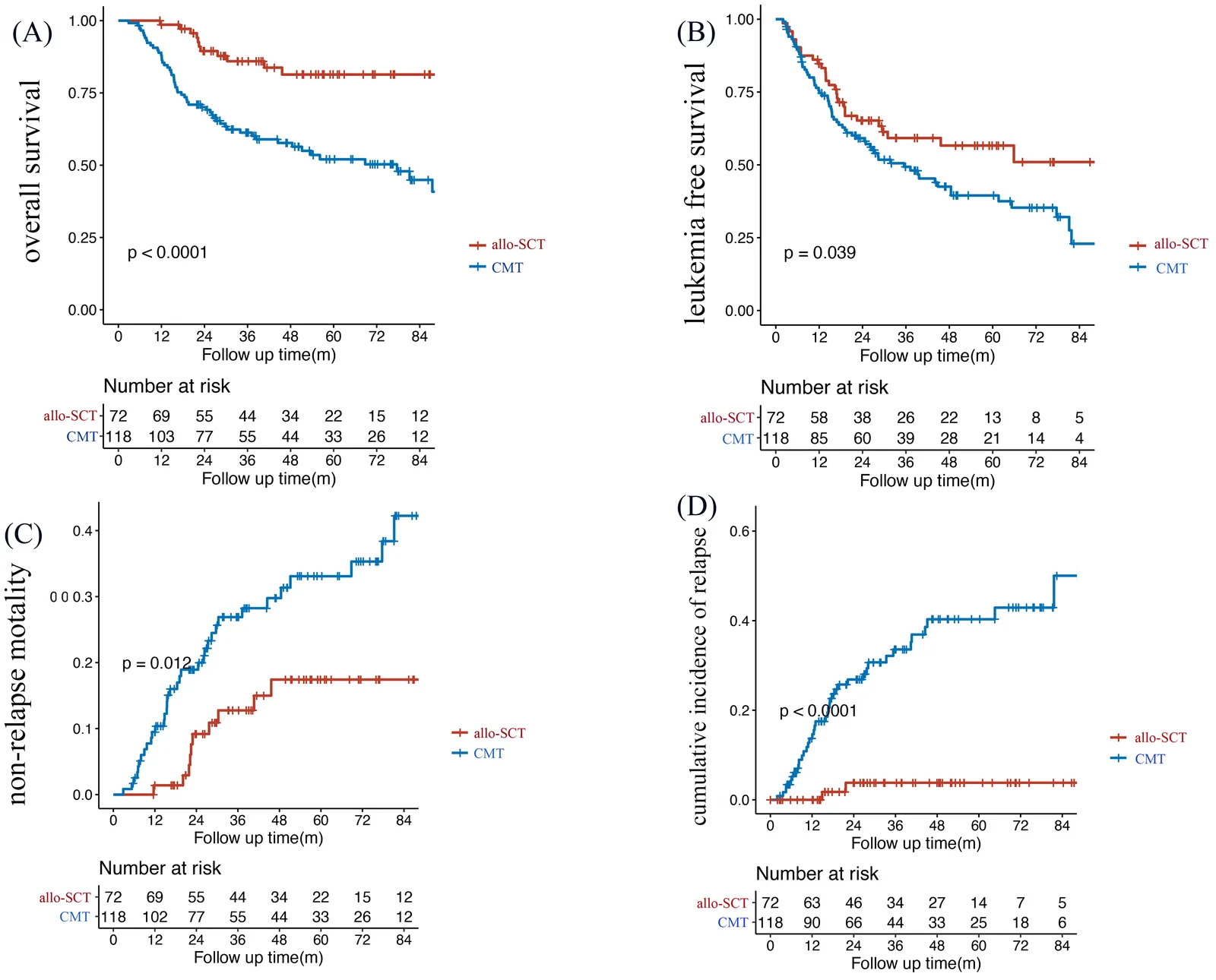
Survival outcomes by treatment cohort. **(A)** Overall survival (OS); **(B)** leukemia free survival (LFS); **(C)** non-relapse mortality (NRM); **(D)** Cumulative of incidence of relapse (CIR).

To further address potential confounding due to baseline imbalances between the allo-HSCT and chemotherapy groups, we performed an IPTW analysis based on propensity scores. After weighting, baseline characteristics were well balanced overall, with standardized mean differences below 0.1 for all but one included variable. In the weighted analysis, allo-HSCT remained associated with superior survival outcomes, and the results were consistent with those of the primary analysis (**Supplementary Figures 1**, **2**).

### Subgroup analyses by disease status

In a stratified survival analysis according to disease status, the 3-year OS was substantially higher with allo-HSCT than with chemotherapy across both CR1-AML and R/R AML patients. Among patients with CR1-AML, allo-HSCT was associated with a significantly superior 3-year OS compared with chemotherapy alone (92.8% vs. 73.6%, *P* = 0.0099; **Figure 3A**). In a sensitivity analysis approximating an intention-to-treat framework(ITT-like), patients in the favorable-risk AML cohort were re-stratified according to HSCT status in CR1. Patients who underwent transplantation only after relapse were not assigned to the CR1-HSCT group and were censored at the time of transplantation. The results were generally consistent with the primary analysis, further supporting the robustness of our findings(92.8% versus 75.2%; *P* = 0.026; **Supplementary Figure 3**). In the R/R AML cohort, overall survival calculated from the date of relapse demonstrated a substantial and statistically significant survival advantage for allo-HSCT recipients compared with chemotherapy-treated patients (73.5% vs. 26.6%; *P* = 0.00015; **Figure 3B**). To further address the potential influence of treatment timing on survival outcomes, a landmark analysis was performed. Consistent with the primary analysis, the allo-HSCT group maintained a significant survival advantage over the chemotherapy group, with OS rates of 78.6% versus 40.7%, respectively (*P* = 0.029; **Supplementary Figure 4**).

**Figure 3:**
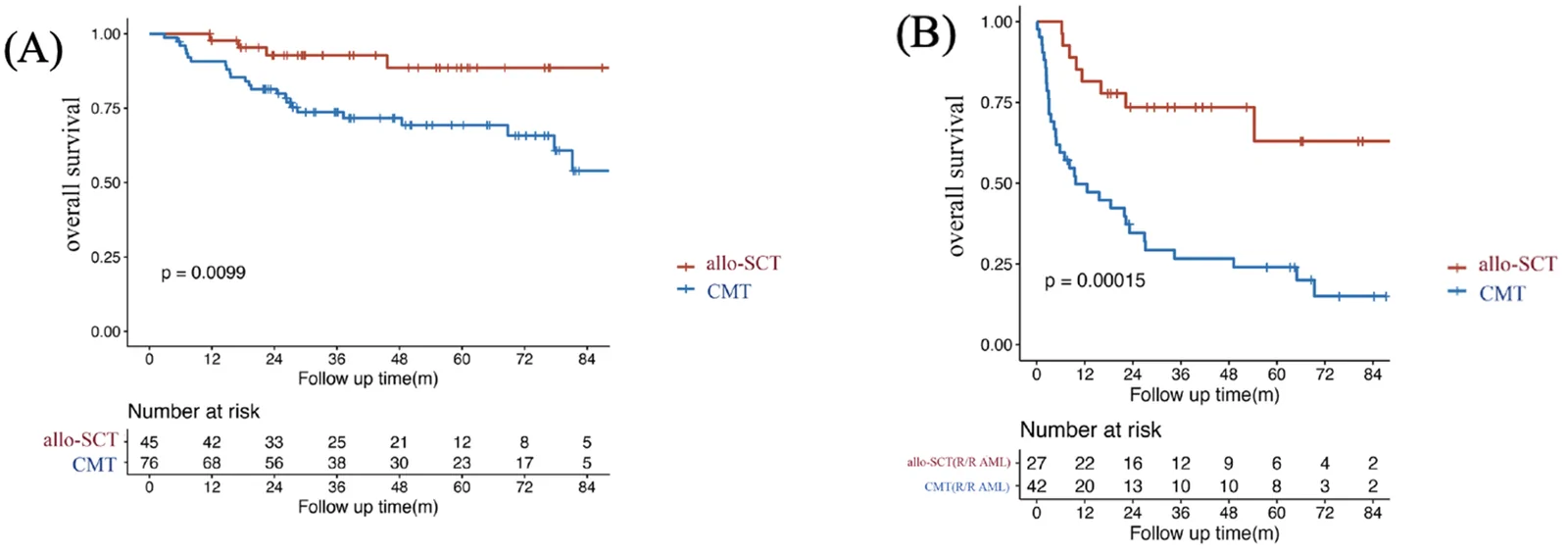
Survival outcomes by disease status and treatment modality **(A)** Overall survival (OS) in patients with CR1-AML treated with allo-HSCT versus chemotherapy alone; **(B)** Overall survival calculated from the date of relapse in R/R AML patients treated with allo-HSCT versus chemotherapy;.

### Subgroup analyses by age

Age-stratified survival analysis demonstrated that patients younger than 20 years with favorable-risk AML had comparable survival after chemotherapy or allo-HSCT, suggesting that allo-SCT may not be necessary for this subgroup. In contrast, among patients aged 20 to 60 years, allo-SCT provided a significant survival advantage over chemotherapy, supporting its use when a suitable donor is available (**Figure 4**).

**Figure 4:**
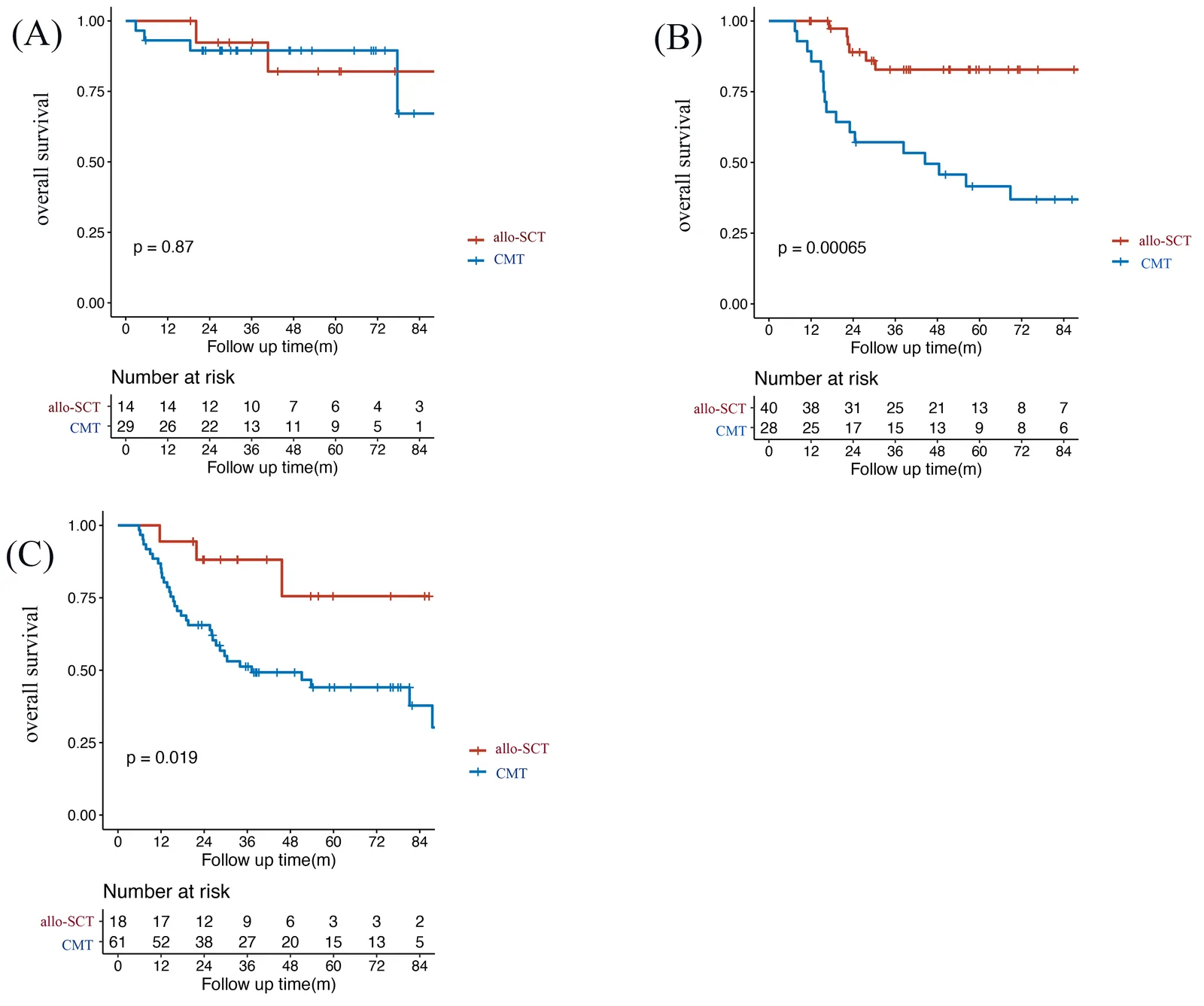
Survival outcomes by age group. **(A)**<20 years; **(B)** 20–40 years; **(C)**> 40 years.

### Subgroup analysis by molecular abnormalities

We conducted subgroup analyses based on different molecular abnormalities to assess the differential impact of allo-HSCT on survival outcomes. The survival benefit of allo-HSCT varied considerably according to the molecular profile. Patients harboring RUNX1::RUNX1T1 fusion or CEBPA bZIP in-frame mutations demonstrated significantly improved three-year OS following allo-HSCT compared with chemotherapy alone (89.1% vs. 48.5%; P < 0.001; 87.1% vs. 68.8%; P = 0.028, respectively; **Figures 5A, D**). By contrast, no meaningful difference in survival was detected between the allo-HSCT and chemotherapy groups among patients with CBFβ::MYH11 fusion (60.6% vs. 64.6%;P = 0.62; **Figure 5B**). Among NPM1-mutated patients, a numerically higher three-year OS was observed in the allo-HSCT group (100% vs. 73.1%; P = 0.16; **Figure 5C**); however, this finding should be interpreted with caution given the limited sample size in this subgroup.

**Figure 5:**
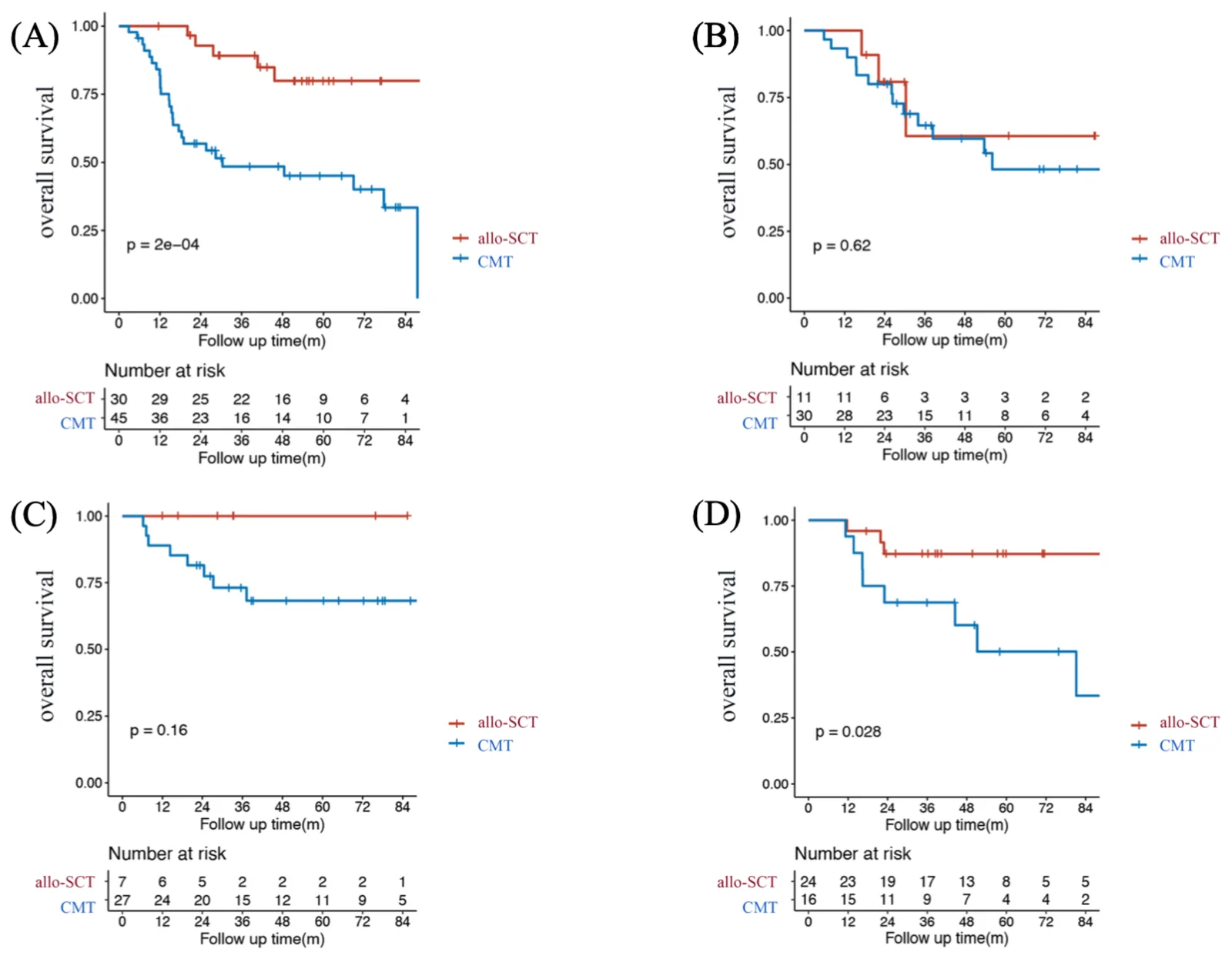
Survival outcomes stratified by molecular abnormalities. Overall survival (OS) was compared between the allo-HSCT and chemotherapy groups in patients with: **(A)** RUNX1::RUNX1T1 fusion; **(B)** CBFβ::MYH11 fusion; **(C)** NPM1 mutation; and **(D)** CEBPA bZIP in-frame mutation.

### Survival analysis of the transplant group

The median age at transplantation was similar between the relapsed/refractory (R/R) AML group and the first complete remission (CR1) AML group (33.4 and 30.9 years, respectively), with no statistically significant difference. However, the proportion of patients with a European Group for Blood and Marrow Transplantation (EBMT) score >2 was significantly higher in the R/R AML group than in the CR1-AML group (81.5% vs. 35.6%; *P* < 0.001), indicating a higher transplantation-risk profile. Regarding engraftment, the median time to neutrophil recovery was 15 days in the R/R AML group and 14 days in the CR1-AML group, whereas the median time to platelet recovery was 18 days and 16 days, respectively. These differences were not statistically significant. No significant differences were observed in donor type, sex mismatch (female donor to male recipient), stem cell source, conditioning regimen, or graft-versus-host disease (GVHD) prophylaxis, suggesting that transplantation protocols were comparable between the groups (**Table 2**).

**Table 2:** The demographic characteristics of patients in allo-SCT group.

Characteristics	CR1-AML (N = 45)	R/R AML (N = 27)	P value
Age at transplantation, median, years	30.9	33.4	0.423
EBMT scores:			<0.001
≤2	29 (64.4%)	5 (18.5%)	
>2	16 (35.6%)	22 (81.5%)	
Female-male, n (%):			0.282
Yes	11 (24.4%)	3 (11.1%)	
No	34 (75.6%)	24 (88.9%)	
Donor type, n (%):			0.924
HRD	2 (4.44%)	1 (3.70%)	
MSD	1 (2.22%)	0 (0.00%)	
CB	19 (42.2%)	14 (51.9%)	
CB+HRD	12 (26.7%)	5 (18.5%)	
CB+MSD	11 (24.4%)	7 (25.9%)	
Source of stem cell, n (%):			0.928
PB	3 (6.67%)	1 (3.70%)	
PB+UCB	23(51.11%)	12 (44.4%)	
UCB	19(42.22%)	14 (51.9%)	
Conditioning regimen, n (%):			0.737
FACyBu	15 (33.3%)	12 (44.4%)	
ACyBu	2 (4.44%)	0 (0.00%)	
FCyBu	7 (15.6%)	2 (7.41%)	
FABuM	1 (2.22%)	0 (0.00%)	
FBuM	19 (42.2%)	13 (48.1%)	
FB2	1 (2.22%)	0 (0.00%)	
GVHD-prevention, n (%):			0.417
CsA+MMF+ATG	11 (24.4%)	3 (11.1%)	
CsA+MMF	6 (13.3%)	5 (18.5%)	
CsA+MMF+PTCY	20 (44.4%)	16 (59.3%)	
CsA+MMF+PTCY+ATG	8 (17.8%)	3 (11.1%)	

EBMT, European Society for Blood and Marrow Transplantation; HRD, Haplotype-Related Donor; MSD, Matched Sibling Donor; CB, Cord Blood; PB, Peripheral Blood; UCB, Unrelated Cord Blood; F, Fludarabine; A, Cytarabine (Ara-C); Cy, Cyclophosphamide; Bu, Busulfan; M, Melphalan; CsA, Cyclosporine A; MMF, Mycophenolate Mofetil; PTCY, Post-Transplant Cyclophosphamide; ATG, Anti-Thymocyte Globulin.

The 3-year OS in the transplant group was 77.0% for R/R AML patients and 92.8% for CR1-AML patients; this difference approached but did not reach statistical significance (P = 0.086; **Figure 6A**). Among patients with pre-transplant MRD data, 3-year OS was 86.0% in MRD-positive cases and 85.6% in MRD-negative cases, with no statistically significant difference (P = 0.35; **Figure 6B**). Similarly, when patients who were MRD-positive or had relapsed before transplantation were compared with patients who were MRD-negative and had not relapsed before transplantation, no statistically significant difference was observed between the groups (84.4% vs 89.9%; P = 0.34, **Figure 6C**). These findings suggest that allo-HSCT may provide substantial survival benefits for both MRD-positive and MRD-negative patients, although larger research is needed to confirm these results.

**Figure 6:**
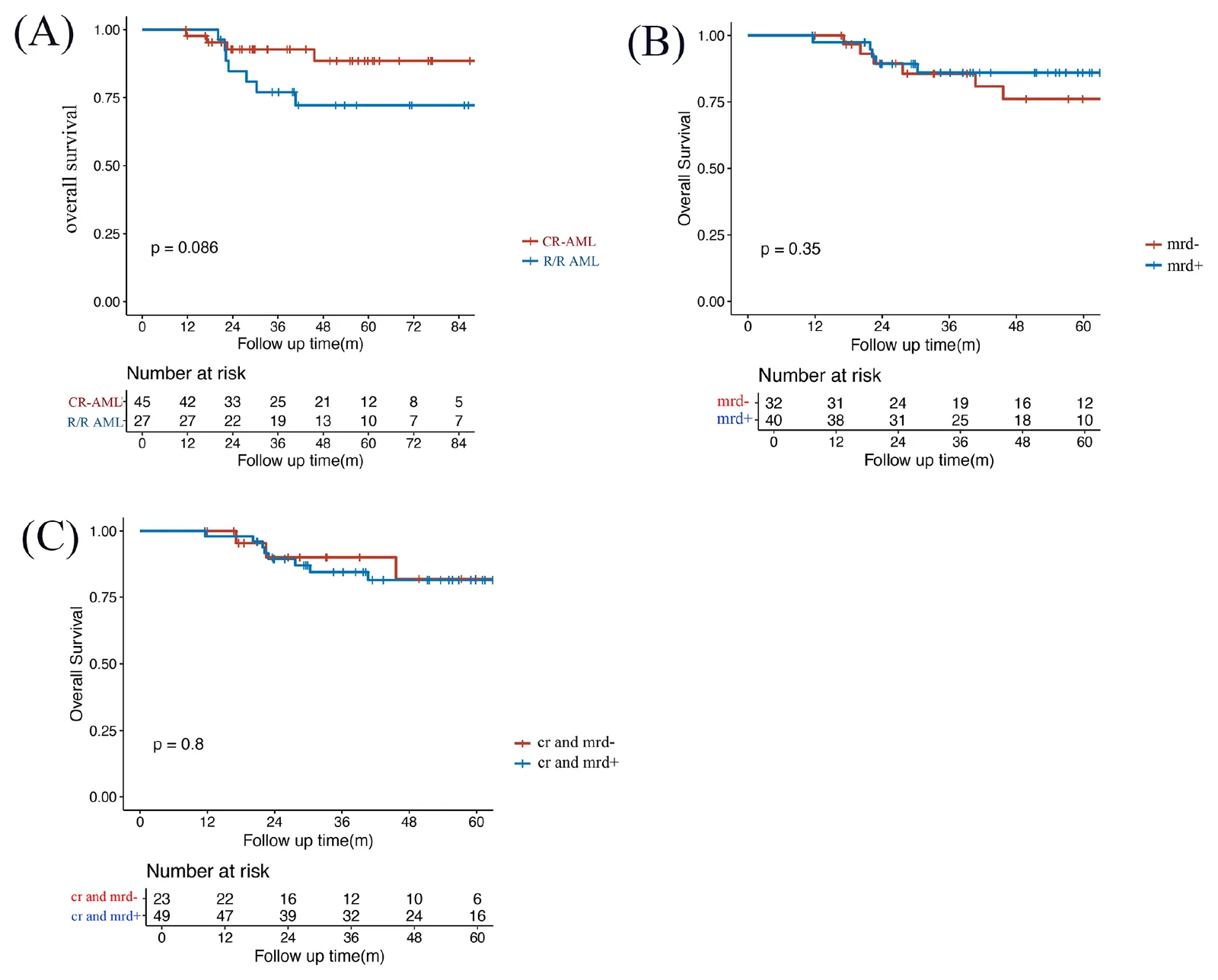
Survival outcomes in the transplant group **(A)** Overall survival (OS) in R/R AML patients and CR1-AML patients; **(B)** OS according to pre-transplant MRD status; **(C)** OS in patients with MRD positivity or relapse before transplantation versus those who were MRD-negative and relapse-free before transplantation.

### Multivariate analysis

The results of the univariable Cox regression analysis are provided in the **Supplementary Materials** (**Supplementary Figures 5**, **6**). Multivariable Cox regression analysis identified chemotherapy, advanced age, and relapsed/refractory disease as independent adverse prognostic factors for OS, and chemotherapy and relapsed/refractory disease as independent adverse prognostic factors for LFS (**Table 3**).

**Table 3:** Multivariate analysis.

Prognostic factors	OS	LFS		
	HR (95%CL)	P value	HR (95%CL)	P valve
Treatment				
Transplant	1.00	–	1.00	–
Chemotherapy	4.34(2.19,8.59)	<0.0001	1.87(1.15, 3.05)	0.01
Age	1.03(1.01,1.04)	0.01	1.00(0.99, 1.02)	0.55
WBC	1.00(1.00,1.01)	0.21	1.00(1.00, 1.01)	0.19
Cycles to CR				
≤2	1.00	–	1.00	–
>2	0.99(0.48,2.03)	0.99	1.26(0.71, 2.23)	0.43
Cycles of chemotherapy	0.97(0.92,1.03)	0.30	0.96(0.92, 1.01)	0.09
R/R AML				
No	1.00	–	1.00	–
Yes	2.53(1.43,4.47)	<0.001	11.44(6.33,20.68)	<0.0001

OS, overall survival; LFS, leukemia-free survival; WBC, White Blood Cell; CR, complete remission; R/R AML, relapsed/refractory acute myeloid leukemia.

## Discussion

In this retrospective study, we compared outcomes of allo-HSCT and chemotherapy in patients with AML harboring favorable-risk genetic features at diagnosis. Allo-HSCT was associated with superior 3-year OS and LFS compared with chemotherapy, with the greatest benefit observed among patients with R/R disease. These findings support consideration of allo-HSCT as a therapeutic option for selected patients in this population, particularly in the salvage setting.

AML is a highly heterogeneous in terms of pathogenesis, genetic characteristics, immunophenotype, and response to treatment. Previous large-scale studies reported a 5-year OS rate of 44% among AML patients younger than 55 years, with risk stratification showing OS rates of 64%, 41%, and 11% in favorable-, intermediate-, and adverse-risk groups, respectively (24). A meta-analysis supported a survival benefit of allo-HSCT in intermediate- and poor-risk patients, but its role in favorable-risk disease remains debated (25). Although favorable-risk patients are typically treated with consolidation chemotherapy, some may also benefit from allo-HSCT (26). Consistent with this evidence, our study suggests that selected patients with AML harboring favorable-risk genetic features at initial diagnosis may achieve improved survival with allo-HSCT, supporting further investigation of transplantation in carefully selected patients. In our cohort, allo-HSCT achieved superior 3-year OS (86.0% vs. 61.3%) and LFS (59.2% vs. 49.3%) compared with chemotherapy, and substantially reduced CIR (3.8% vs. 33.6%). These findings suggest that allo-HSCT may be associated with improved survival and a lower risk of relapse in selected patients with FR-AML.

Relapsed or refractory AML remains difficult to treat and is associated with high mortality. High-dose cytarabine combined with mitoxantrone is a commonly used regimen in this setting (27). However, outcomes remain suboptimal, and the role of allo-HSCT continues to be investigated (28, 29). In a recent study evaluating pre-transplant treatment strategies, only patients with favorable-risk AML demonstrated superior OS with a cytoreduction-based approach in exploratory analyses. Notably, most favorable-risk patients in that study were enrolled with untreated morphologic relapse. Although the authors cautioned that this subgroup finding should be interpreted carefully and might have been incidental, favorable-risk AML was still associated with an approximately 66% 5-year OS after allo-HSCT, substantially higher than the approximately 34% observed in adverse-risk AML. These findings underscore the persistent prognostic importance of AML genetics even in the post-transplant setting (30). Similarly, one of our objectives was to investigate whether patients initially classified as FR-AML who subsequently developed refractory or relapsed disease could still derive meaningful survival benefit from salvage allo-HSCT. Among patients in first complete remission (CR1-AML), 3-year OS was substantially higher following allo-HSCT than with chemotherapy alone (92.8% vs. 73.6%), suggesting a potential survival benefit of allo-HSCT in selected CR1 patients within this cohort. In R/R AML, allo-HSCT was also associated with markedly better survival than chemotherapy (73.5% vs. 26.6%), demonstrating that salvage transplantation can achieve meaningful long-term survival even in the setting of relapse or refractory disease. Importantly, the landmark analysis produced findings that were in line with the main results, showing that allo-HSCT remained associated with significantly improved OS compared with chemotherapy alone. The consistency between the landmark analysis and the main analysis supports the robustness of the observed association, although residual bias related to treatment classification and timing cannot be excluded. Nevertheless, given the retrospective design and limited sample size of the present study, these findings should still be interpreted with caution and warrant further validation in larger prospective studies. These findings address a critical clinical question: whether FR-AML patients who develop relapse or refractory disease can still benefit from delayed allo-HSCT. Our results demonstrate that the 3-year OS after allo-HSCT was numerically lower in patients transplanted in the R/R AML setting than in those transplanted in the CR1-AML setting (77.0% vs. 92.8%), but the difference did not reach statistical significance. This finding suggests that delayed transplantation may still be a viable strategy for patients initially diagnosed with FR-AML who subsequently relapse. This observation supports the feasibility of deferred allo-HSCT in selected cases, highlighting its potential as a viable therapeutic strategy even after disease relapse.

Age-stratified analysis revealed comparable outcomes between chemotherapy and allo-HSCT in younger (<20 years) patients with AML harboring favorable-risk genetic features at initial diagnosis, suggesting no clear survival advantage of allo-HSCT over chemotherapy in this subgroup. In contrast, allo-HSCT was associated with better survival in patients aged 20–60 years. These findings underscore the importance of incorporating age into treatment selection and support a personalized approach to post-remission therapy in AML.

Molecular subgroup analyses suggested heterogeneity in the association between allo-HSCT and survival across genetic subgroups. Patients with RUNX1::RUNX1T1 and CEBPA bZip in-frame mutation showed the most pronounced survival differences between the treatment groups, whereas CBFβ::MYH11 patients did not show a clear advantage. Although NPM1-mutated patients showed numerically higher survival with allo-HSCT, small sample size limits interpretation. These results underscore the importance of a mutation-informed, individualized approach to transplant decision-making.

Advancements in detection methods now allow MRD to be identified below the 5% bone marrow blast threshold, making MRD status a critical factor in post-remission treatment decisions (31–33). In our cohort, MRD data were more complete in the transplant group, allowing analysis of 66 patients with available pre-transplant MRD data. The 3-year OS rates in the MRD-positive and MRD-negative groups were 86.0% and 85.6%, respectively, with no statistically significant difference (P = 0.35). These findings raise the possibility that allo-HSCT may mitigate the adverse prognostic impact associated with MRD positivity. However, given the limited sample size and statistical power of this subgroup analysis, this observation should be interpreted cautiously and considered hypothesis-generating rather than definitive. Further validation in larger cohorts with standardized MRD monitoring is warranted.

The primary limitation of this study is the selection bias inherent to its retrospective design. Treatment allocation was not randomized, and patients who underwent allo-HSCT represented a clinically selected subgroup rather than an unselected favorable-risk AML population. Although we performed IPTW and landmark analyses, including an intent-to-treat-like treatment classification, to reduce potential bias and to test the robustness of the results, residual bias cannot be completely eliminated in a retrospective study. The intent-to-treat-like classification used in the landmark analysis may have introduced treatment-exposure misclassification, as some patients assigned to the allo-HSCT group may not have ultimately undergone transplantation, potentially attenuating the estimated effect of allo-HSCT. Accordingly, the observed survival benefit associated with allo-HSCT should not be generalized to all patients with favorable-risk AML or interpreted as evidence supporting routine transplantation in this population, nor should it be viewed as contradicting current ELN recommendations. Center-specific practices may limit the generalizability of these findings, highlighting the need for further multicenter studies. Additionally, MRD status, a critical prognostic factor in AML, was not uniformly available due to incomplete recording during the study period. Inclusion of MRD in multivariate analyses would have substantially reduced the analyzable sample size and potentially introduced selection bias. Consequently, MRD was not incorporated into the analysis. We acknowledge this limitation and emphasize the need for prospective studies with standardized MRD monitoring to better guide treatment decisions between chemotherapy and allo-HSCT. Therefore, our findings should be considered hypothesis-generating rather than definitive. Despite the use of multiple statistical approaches to mitigate bias, prospective clinical trials or carefully designed multicenter studies are still required to confirm the role of allo-HSCT in selected patients with AML harboring favorable-risk genetic features at initial diagnosis.

## Conclusion

Allo-HSCT was associated with improved OS and LFS and a lower risk of relapse compared with chemotherapy in patients with AML harboring favorable-risk genetic features at initial diagnosis and reduced both non-relapse mortality and relapse rates. These benefits were observed not only in patients who achieve complete remission prior to transplantation, but also in those who develop relapsed or refractory disease. These findings support salvage allo-HSCT as a potential therapeutic option for selected patients with AML harboring favorable-risk genetic features at initial diagnosis who relapse, and suggest that deferred transplantation may be feasible in carefully selected patients with an available suitable donor. For younger patients (<20 years), chemotherapy may suffice. Given the retrospective and non-randomized nature of this study, these findings should be considered hypothesis-generating rather than definitive. Prospective clinical trials are warranted to validate these findings, establish optimal transplantation timing, and refine post-remission treatment guidelines for favorable-risk AML across different disease stages.

## Data Availability

The raw data supporting the conclusions of this article will be made available by the authors, without undue reservation.
