# Supplementary material for: Allogeneic hematopoietic stem cell transplantation is associated with improved survival compared with chemotherapy in patients with acute myeloid leukemia harboring favorable-risk genetic features at initial diagnosis: a single-center retrospective study
Source: Front Immunol. 2026 Sep 15;17:1825310. doi: 10.3389/fimmu.2026.1825310 (PMC13619477; doi:10.3389/fimmu.2026.1825310)
Supplement: Supplementary file 1 [file SupplementaryFile1.pdf]

Supplementary Figure S1

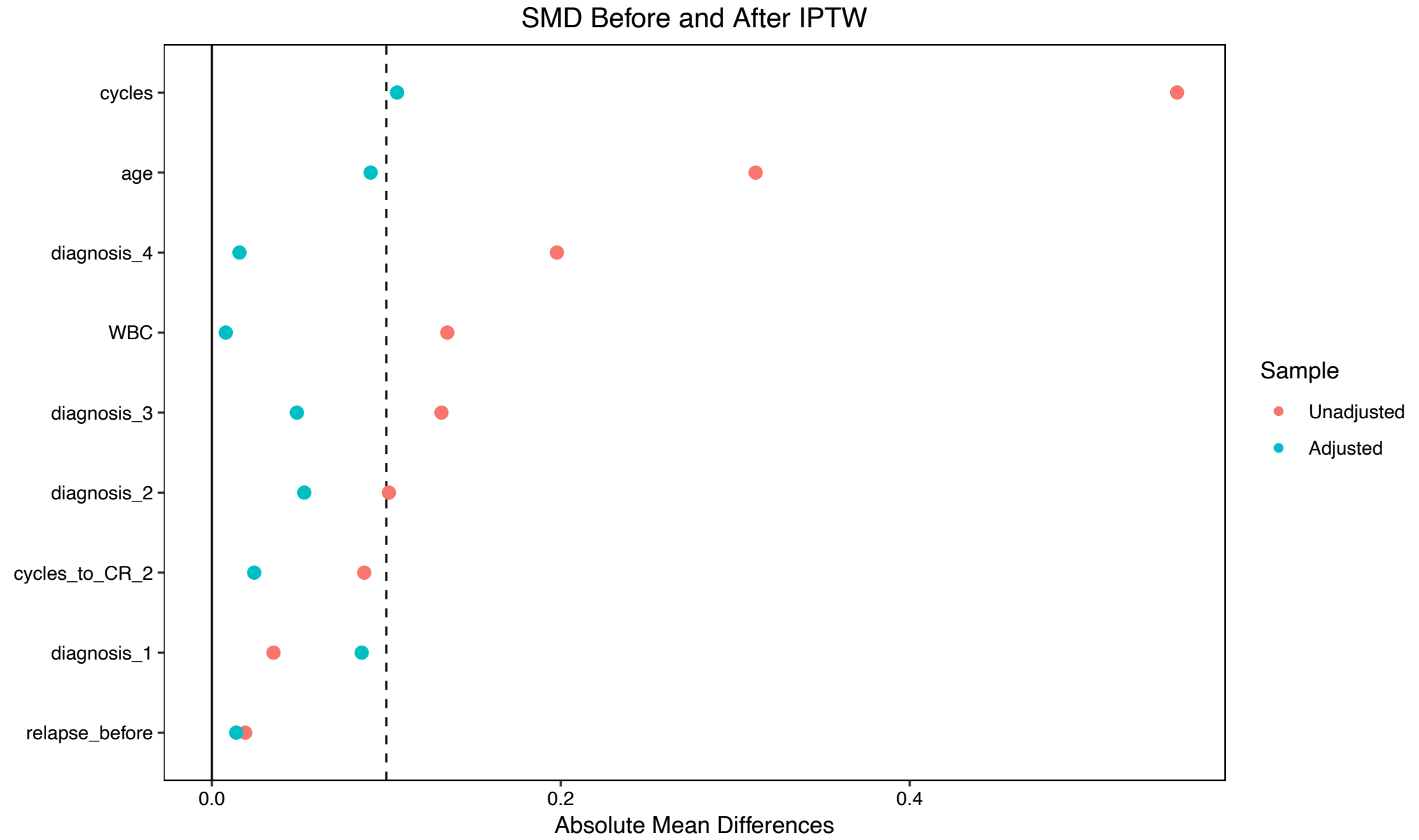

Figure S1: SMD before and after IPTW.

Supplementary Figure S2

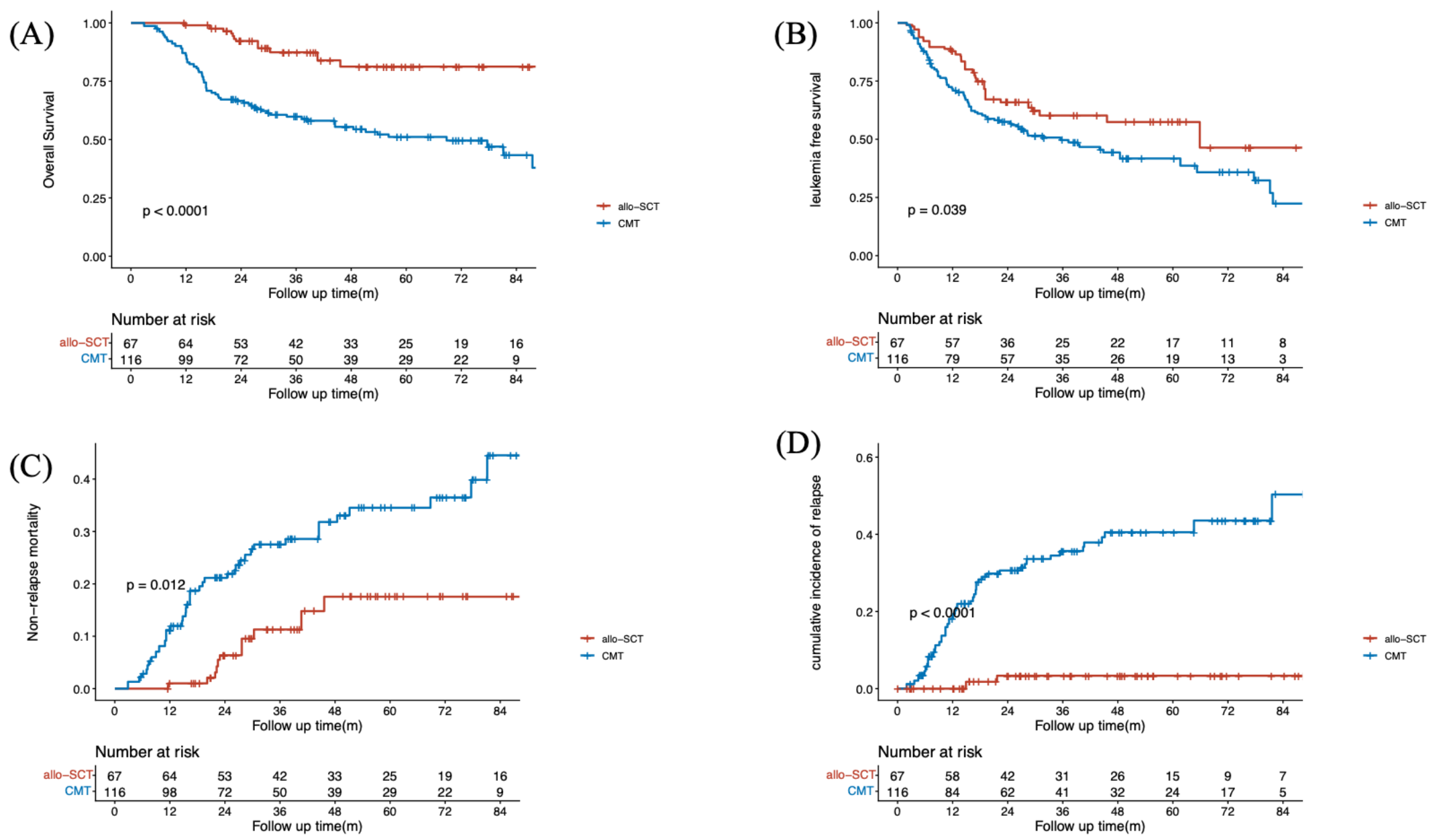

Figure S2: Survival outcomes after IPTW. (A) Overall survival (OS); (B) leukemia free survival (LFS); (C) non-relapse mortality (NRM); (D) Cumulative of incidence of relapse (CIM).

Supplementary Figure S3.

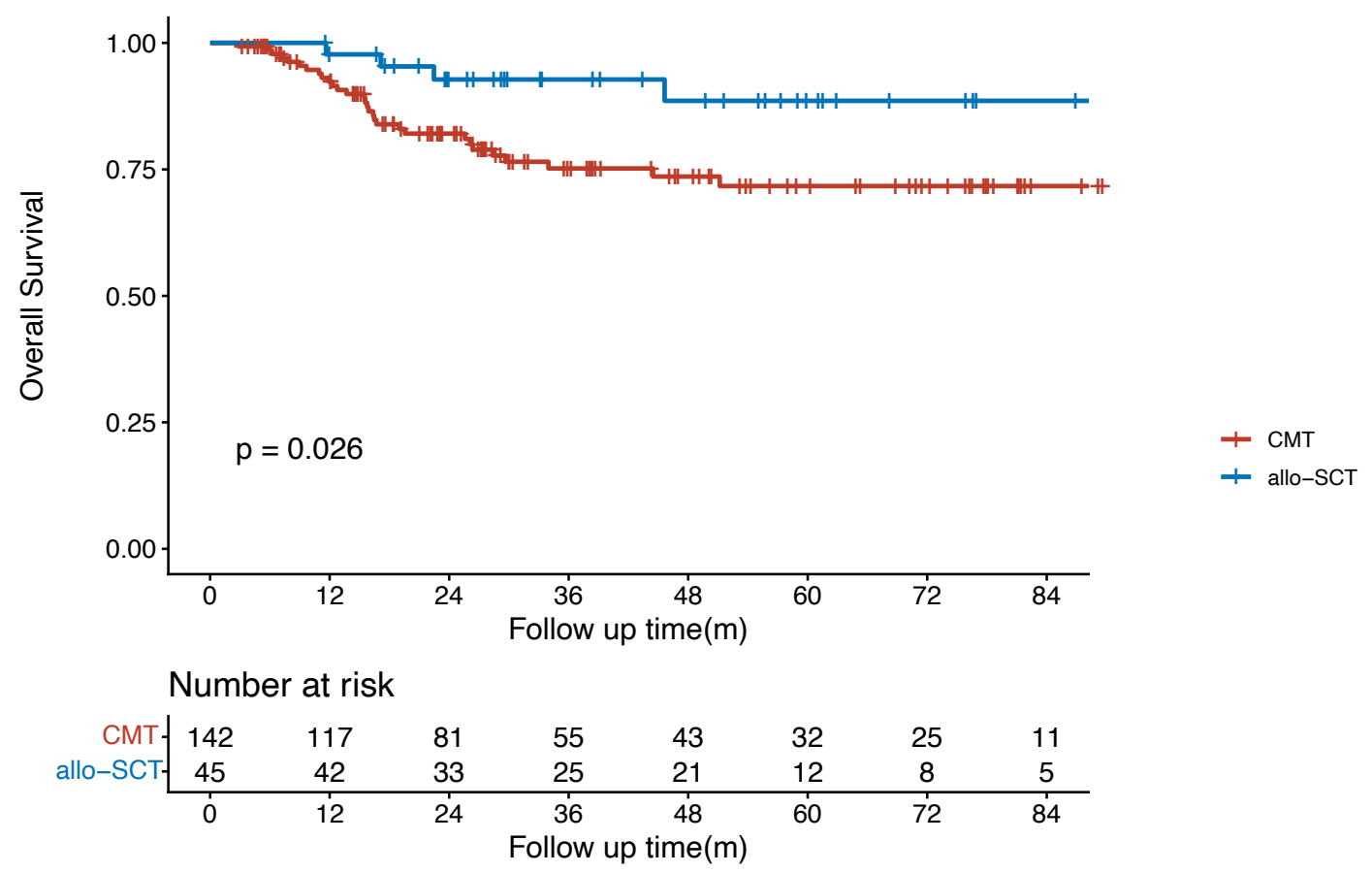

Supplementary Figure S3. intention-to-treat like framework: Overall survival of patients re-stratified according to HSCT status in CR1.

Supplementary Figure S4.

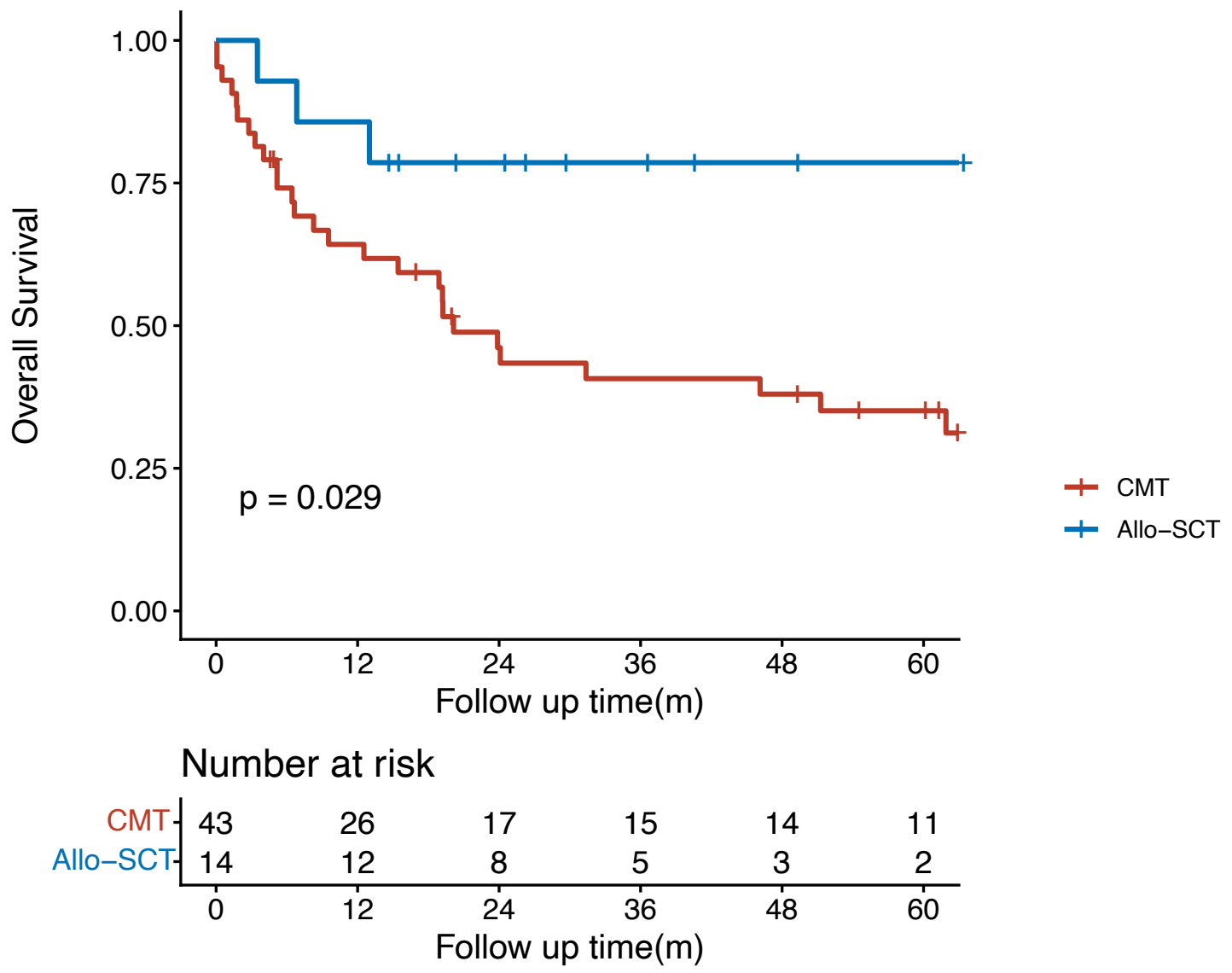

Figure S4 Landmark analysis in R/R AML cohort.

Supplementary Figure S5.

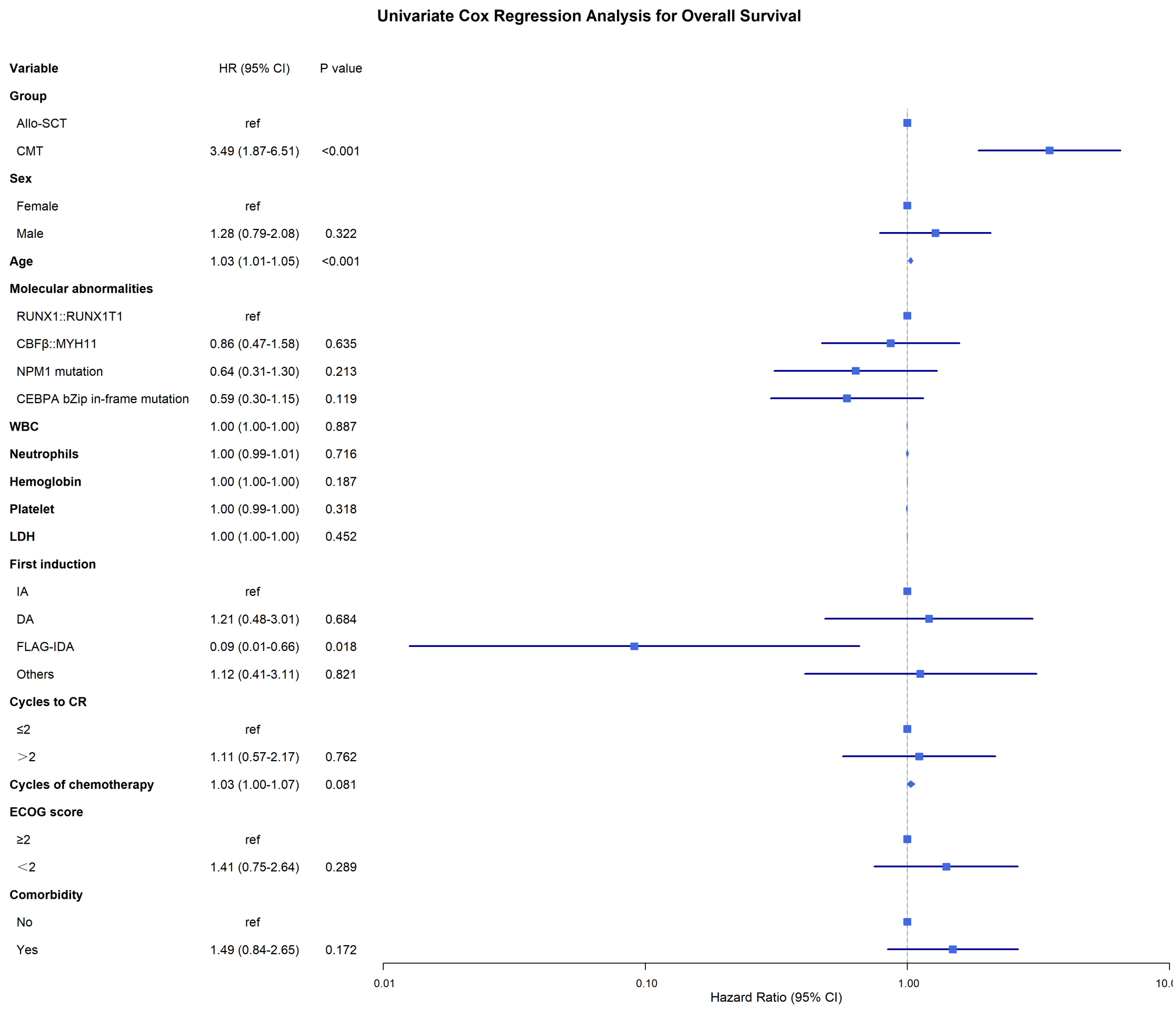

Figure S5. Univariate Cox Regression Analysis for Overall Survival.

Supplementary Figure S6.

Univariate Cox Regression Analysis for LFS

Variable	HR (95% CI)	P value
**Group**		
Allo-SCT	ref	
CMT	1.57 (1.02-2.43)	0.041
**Sex**		
Female	ref	
Male	0.87 (0.58-1.30)	0.496
**Age**	1.02 (1.01-1.04)	<0.001
**Molecular abnormalities**		
RUNX1::RUNX1T1	ref	
CBFβ::MYH11	0.90 (0.52-1.55)	0.711
NPM1 mutation	0.73 (0.41-1.30)	0.286
CEBPA bZip in-frame mutation	0.89 (0.52-1.52)	0.675
**WBC**	1.00 (1.00-1.00)	0.904
**Neutrophils**	1.00 (0.99-1.01)	0.508
**Hemoglobin**	1.00 (1.00-1.00)	0.586
**Platelet**	1.00 (0.99-1.00)	0.123
**LDH**	1.00 (1.00-1.00)	0.555
**First induction**		
IA	ref	
DA	1.12 (0.52-2.43)	0.778
FLAG-IDA	0.15 (0.05-0.47)	0.001
Others	0.85 (0.31-2.32)	0.747
**Cycles to CR**		
≤2	ref	
＞2	1.77 (1.05-2.99)	0.034
**Cycles of chemotherapy**	1.07 (1.04-1.10)	<0.001
**ECOG score**		
≥2	ref	
＜2	1.27 (0.74-2.18)	0.391
**Comorbidity**		
No	ref	
Yes	1.32 (0.80-2.16)	0.275

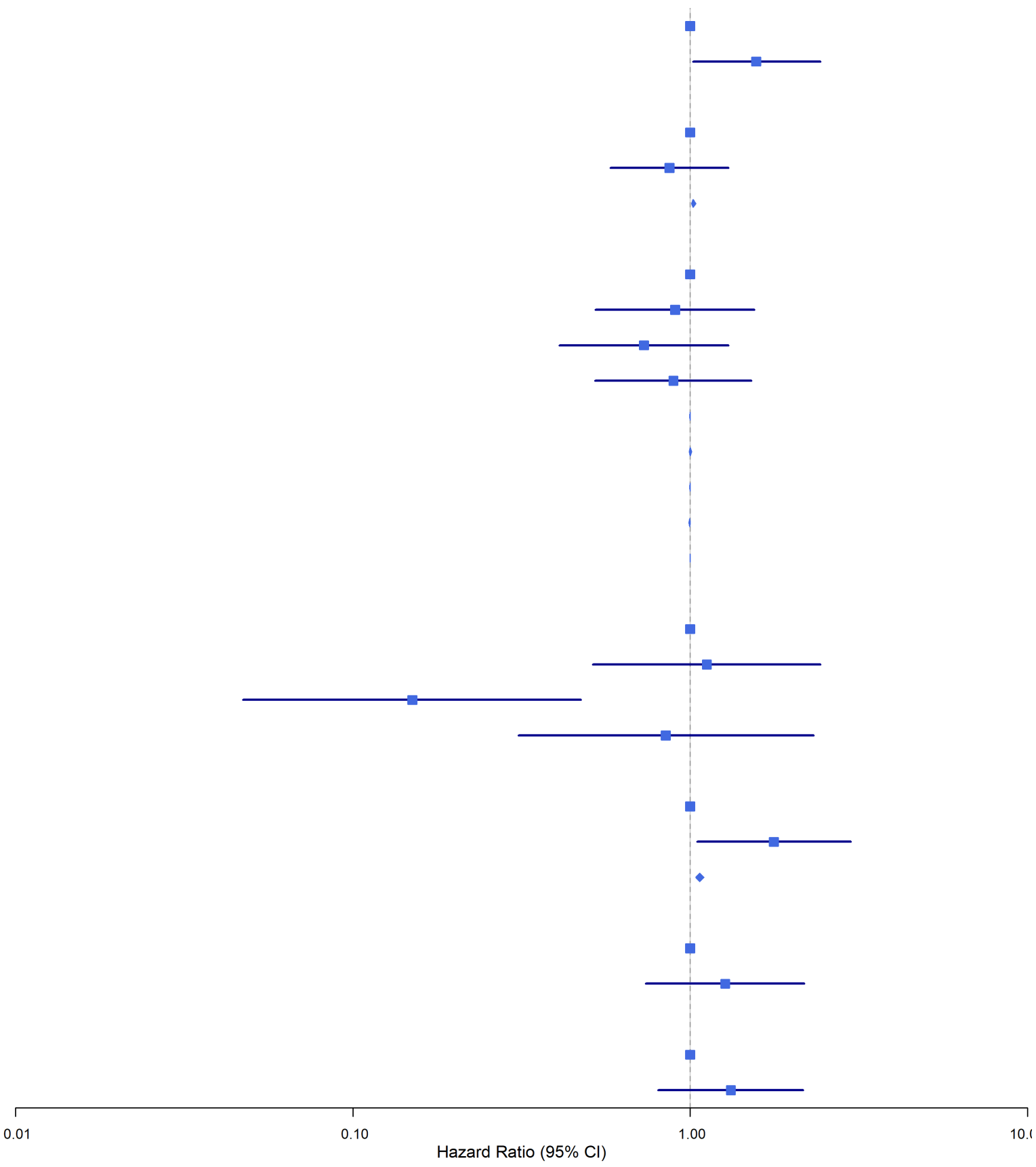

Figure S6. Univariate Cox Regression Analysis for LFS.
