# Supplementary material for: Allogeneic hematopoietic stem cell transplantation is associated with improved survival compared with chemotherapy in patients with acute myeloid leukemia harboring favorable-risk genetic features at initial diagnosis: a single-center retrospective study
Source: Front Immunol. 2026 Sep 15;17:1825310. doi: 10.3389/fimmu.2026.1825310 (PMC13619477; doi:10.3389/fimmu.2026.1825310)
Supplement: Supplementary file 2 [file SupplementaryFile2.pdf]

Supplementary Table 1. MRD detection methods and threshold used in the study cohort

MRD modality	Target/assessment	Detection method	Threshold
Flow cytometric MRD	Residual leukemic cells with a leukemia-associated immunophenotype (LAIP)	Multiparameter flow cytometry (MFC)	Residual leukemic cells $>10^{-4}$
Molecular MRD	RUNX1::RUNX1T1	Real-time quantitative PCR (qPCR)	Fusion transcript/reference gene ratio ≥0.01%
Molecular MRD	CBFB::MYH11	Real-time quantitative PCR (qPCR)	Fusion transcript/reference gene ratio ≥0.01%
Molecular MRD	NPM1 mutation	Digital PCR	Mutant allele fraction ≥0.01%
Molecular MRD	CEBPA bZip in-frame mutation	Digital PCR	Mutant allele fraction ≥0.01%
